# Oxidative Stress Markers in Hypertrophic Cardiomyopathy

**DOI:** 10.3390/medicina58010031

**Published:** 2021-12-24

**Authors:** Bożena Szyguła-Jurkiewicz, Wioletta Szczurek-Wasilewicz, Tadeusz Osadnik, Anna M. Frycz-Kurek, Karolina Macioł-Skurk, Justyna Małyszek-Tumidajewicz, Michał Skrzypek, Ewa Romuk, Mariusz Gąsior, Maciej Banach, Jacek J. Jóźwiak

**Affiliations:** 1 3rd Department of Cardiology, School of Medical Sciences in Zabrze, Medical University of Silesia in Katowice, 40-055 Katowice, Poland; centrala4@wp.pl (B.S.-J.); m.gasior@op.pl (M.G.); 2 Silesian Center for Heart Diseases, 3rd Department of Cardiology, 41-800 Zabrze, Poland; tadeusz.osadnik@sccs.pl (T.O.); annaf@hot.pl (A.M.F.-K.); k.maciol.skurk@gmail.com (K.M.-S.); 3 Department of Pharmacology, Faculty of Medical Sciences in Zabrze, Medical University of Silesia in Katowice, 40-055 Katowice, Poland; 4 Department of Cardiac, Vascular and Endovascular Surgery and Transplantology in Zabrze, Silesian Center for Heart Diseases, 41-800 Zabrze, Poland; jmalyszek@gmail.com; 5 Department of Biostatistics, School of Public Health in Bytom, Medical University of Silesia in Katowice, 40-055 Katowice, Poland; mskrzypek@sum.edu.pl; 6 Department of Biochemistry, School of Medical Sciences in Zabrze, Medical University of Silesia in Katowice, 40-055 Katowice, Poland; eromuk@sum.edu.pl; 7 Polish Mothers Memorial Hospital Research Institute, 90-419 Łódź, Poland; maciej.banach@umed.lodz.pl; 8 Department of Hypertension, Chair of Nephrology and Hypertension, Medical University of Lodz, 90-419 Łódź, Poland; 9 Cardiovascular Research Centre, University of Zielona Gora, 65-046 Zielona Gora, Poland; 10 Department of Family Medicine and Public Health, Faculty of Medicine, University of Opole, 45-040 Opole, Poland; jacek.jozwiak.1234@gmail.com

**Keywords:** oxidative stress, markers, hypertrophic cardiomyopathy

## Abstract

*Background and Objectives*: Hypertrophic cardiomyopathy (HCM) depends on the primary impairment of sarcomeres, but it can also be associated with secondary alterations in the heart related to oxidative stress. The present study aimed to examine oxidative-antioxidant disturbances in patients with HCM compared with control individuals. *Materials and Methods*: We enrolled 52 consecutive HCM patients and 97 controls without HCM. The groups were matched for age, body mass index, and sex. Peripheral blood was collected from all patients to determine the total antioxidant capacity (TAC), total oxidant status (TOS), lipid hydroperoxide (LPH), and malondialdehyde (MDA). The oxidative stress index (OSI) was defined as the ratio of the TOS level to the TAC level. *Results*: The median age was 52 years, and 58.4% were female. The area under the curve (AUC) indicated good predictive power for the TAC and TOS [AUC 0.77 (0.69–0.84) and 0.83 (0.76–0.90), respectively], as well as excellent predictive power for the OSI [AUC 0.87 (0.81–0.93)] for HCM detection. Lipid peroxidation markers also demonstrated good predictive power to detect HCM patients [AUC_LPH_ = 0.73, AUC_MDA_ = 0.79]. *Conclusions*: The TOS, the TAC, LPH levels, and MDA levels have good predictive power for HCM detection. The holistic assessment of oxidative stress by the OSI had excellent power and could identify patients with HCM.

## 1. Introduction

Hypertrophic cardiomyopathy (HCM) is a relatively common autosomal dominant cardiovascular disorder. It is characterized by diverse clinical and phenotypic presentations, ranging from asymptomatic mutation carriers to severely symptomatic patients with a risk of sudden cardiac death or left ventricular outflow obstruction, which could be associated with the development of heart failure in this group of patients [1,2,3]. The major underlying structural abnormalities in HCM, such as coronary microvasculature dysfunction, myocardial disarray, and remodeling changes, lead to impaired coronary reserve, diastolic dysfunction, and arrhythmias [4,5,6]. Genetic testing in HCM patients shows many identified sarcomere mutations. However, it must be emphasized that genotype-phenotype associations are often inconsistent, and sarcomere mutations are unreliable in predicting prognosis [1,2,3]. Thus, the management and risk stratification of HCM patients is based mainly on clinical criteria [4]. HCM is defined as the presence of left ventricular hypertrophy without chamber dilation in the absence of another cardiac, metabolic, or systemic disease [7].

HCM is caused by mutations in sarcomere proteins. However, the pathophysiological mechanisms of HCM are more complex than sarcomere gene mutations alone. First, these mutations cause HCM only in some people. Furthermore, there is evidence that HCM can also be associated with other genetic and nongenetic causes (e.g., mitochondrial or neuromuscular diseases) [8]. Moreover, HCM typically develops in young persons (including trained athletes), while sarcomere gene mutations are present from birth. The clinical, pathological, and genetic heterogeneity of HCM makes the diagnosis of the patients difficult. Thus, early detection of HCM is very important because most patients with HCM are asymptomatic or mildly symptomatic until sudden cardiac death occurs.

Oxidative stress is essentially an imbalance between an increase in the formation of reactive oxygen species (ROS) and the ability of cells to remove or neutralize ROS by antioxidant systems [9]. Under conditions of prolonged exposure of cells to free radicals, antioxidant mechanisms are gradually depleted, which plays a crucial role in the initiation of cell damage and the subsequent cascade of changes [9,10]. The deleterious effects of ROS are mainly due to the ability of ROS to produce changes in subcellular organelles, induce intracellular Ca^2^^+^ overload, and modulate intracellular enzyme activity and gene expression [10,11]. ROS also have the ability to interact with proteins, lipids, and genetic material of the nucleus and mitochondria. These interactions contribute to changes in their conformation, folding, aggregation and cell membrane integration and, as a consequence, the impairment of normal cell function [11,12,13]. From a pathophysiological point of view, oxidative stress may play an important role in HCM. Previous studies have indicated that the development and progression of HCM depends on the primary impairment of sarcomeres caused by the mutation, but it can also be associated with secondary alterations in the heart related to increased oxidative stress [14,15,16]. Oxidative stress may contribute to the intensification of mutations in sarcomere proteins. In turn, mutant sarcomere proteins are a further source of ROS, which act as signaling molecules. ROS stimulate the activation of signal kinases and transcription factors that modify the function of important cellular proteins and signaling pathways in the heart, consequently contributing to the hypertrophic remodeling of the heart [2,17,18]. ROS also damage phospholipid-rich mitochondrial membranes and induce mitochondrial oxidative stress, which are important molecular mechanisms contributing to HCM development and progression [8]. 

Taking into account the potential relationship between oxidative stress and HCM, the aim of our study was to assess oxidative-antioxidant imbalances in patients with HCM compared with control individuals. 

## 2. Material and Methods

### 2.1. Study Population

The analyzed population comprised 52 consecutive patients with hypertrophic cardiomyopathy who had been admitted to our institution between 2018 and 2019. Exclusion criteria comprised infectious diseases, liver and kidney diseases, endocrine disorders, connective tissue diseases, alcohol abuse, and known antioxidant supplementation. The control group included 97 subjects without HCM matched for age, body mass index (BMI), and sex; they were recruited from the LIPIDOGEN2015 substudy between 2015 and 2016 [19]. 

HCM was diagnosed according to the current ESC guidelines for the diagnosis and management of hypertrophic cardiomyopathy, including typical clinical, electrocardiographic, and echocardiographic features [20]. Moreover, at the time of HCM diagnosis, patients underwent cardiac MRI to confirm the diagnosis and exclude other causes of hypertrophy [20]. Coronary angiography was performed only in patients with typical chest pain or risk factors for coronary artery disease at least 6 months prior to study enrollment. 

At the initiation of the study, all HCM patients underwent a physical examination, a panel of laboratory tests, an electrocardiographic examination, an echocardiographic examination, and a 6-min walk test. Patients in the control group underwent a physical examination and a detailed medical interview that collected data on chronic diseases and their treatment, diet, physical activity, smoking status, and family history of disease [19]. For all patients, anthropometric measurements were made (height and body weight without heavy clothing or shoes). BMI was calculated based on height measurements in meters and body mass measurements in kilograms [kg/m^2^]. At the same time, measurements of blood pressure and heart rate were taken. Moreover, 5-mL samples of peripheral blood were collected from all patients to determine the oxidative-antioxidative balance parameters.

The study was approved by the Bioethical Committee of the Medical University of Silesia (specific ethics code—KNW/0022/KB1/102/18, date of approval: 11 December 2018 and by the Bioethical Committee of the Chamber of Physicians (specific ethics code—KBCz-0018/2015, date of approval: 2 December 2015). The study was conducted according to the criteria set by the Declaration of Helsinki, and each patient signed an informed consent form before participating in the study.

### 2.2. Echocardiographic Study

Transthoracic echocardiographic examinations were performed using a Philips Sonos 7500 (Philips Medical Systems, Eindhoven, The Netherlands). M-mode echocardiograms and two-dimensional echocardiograms followed by pulsatile and continuous wave Doppler records were obtained in each patient. Conventional techniques were used to measure the left ventricular size. The left ventricular outflow tract (LVOT) gradient was measured by Doppler recording, and a value > 30 mmHg was considered a clinically relevant cutoff value.

### 2.3. Biochemical Measurements

Blood samples were obtained after 12 h or more of fasting. Standard laboratory tests were measured as soon as possible. The complete blood count and hematologic parameters of patients were analyzed automatically using blood cell counters (Sysmex XS1000i and XE2100, Sysmex Corporation, Kobe, Japan). Liver and kidney function parameters, as well as cholesterol and albumin plasma concentrations, were determined with a COBAS Integra 800 analyzer (Roche Instrument Center AG, Rotkreuz, Switzerland). A highly sensitive latex-based immunoassay was used to detect plasma C-reactive protein with a Cobas Integra 70 analyzer (Roche Diagnostics, Ltd., (Mannheim, Germany)). The plasma concentration of the N-terminal prohormone of brain natriuretic peptide was measured with a commercially available kit from Roche Diagnostics on an Elecsys 2010 analyzer.

For oxidative-antioxidative parameter measurements, a 5-mL blood sample was additionally collected into a plastic tube containing potassium EDTA. Serum samples were centrifuged at 2000× *g* for 10 min and stored in aliquots at −80 °C until biochemical examination. The serum total oxidant status (TOS) and the serum total antioxidant capacity (TAC) levels were determined by the methods described by Erel [21,22]. To evaluate the global balance of oxidation-antioxidation, we calculated the oxidative stress index (OSI), which was defined as the ratio of the TOS level to the TAC level. The lipid hydroperoxide (LHP) concentration in serum was determined by the method of Arab et al. [23]. Malondialdehyde (MDA) concentration was measured according to the method described by Ohkawa et al. [24].

### 2.4. Statistical Analysis

The statistical analysis was performed using SAS software, version 9.4 (SAS Institute Inc., Cary, NC, USA). Continuous data are presented as the mean ± standard deviation for normal distribution, as the median (25th–75th percentile) for nonparametric distribution, and as a percentage for categorical variables. Differences between groups were assessed with the t test for normally distributed data, the Mann–Whitney test for nonnormally distributed continuous variables, and the χ^2^ test for categorical variables. A receiver operating characteristic (ROC) curve was created to determine the utility of the oxidative-antioxidative parameters for HCM detection. The Youden index was used to determine the optimal cutoff value for each biomarker. The prognostic strength of the factors was evaluated by the area under the curves (AUC) from the ROC analysis, as well as their sensitivities, specificities, negative predictive values (NPV), positive predictive values (PPV), negative likelihood ratios (LR−), positive likelihood ratios (LR+), and accuracies. An AUC > 0.7 was considered clinically relevant. Spearman’s rank correlation was performed for the analysis of correlations between oxidative stress parameters for all participants. Values of *p* < 0.05 were considered to be significant. 

## 3. Results

The analyzed population consisted of 52 patients with HCM (median age 52.5 years, 57.7% females) and 97 control subjects (median age 52.0 years, 58.8% females). The control subjects and HCM patients were matched for sex, age, and BMI. There were no differences in the frequency of smoking or the presence of hypertension and hypercholesterolemia in the control group compared with the HCM group. HCM patients had significantly increased levels of oxidative stress markers (TOS, OSI, LPH, and MDA) and decreased total antioxidant levels compared to the control individuals, as defined by TAC. The baseline characteristics of the patients divided into control and HCM groups are presented in Table 1. The concentrations of oxidative stress markers in control and HCM groups are shown in Table 2.

Among HCM patients, 44.2% were found to have left ventricular outflow tract obstruction. There were no differences in the analyzed parameters between HCM patients with LVOT obstruction and those without LVOT obstruction. All patients had asymmetric septal hypertrophy. Alcohol septum ablation was performed in four HCM patients, while two patients underwent septal myectomy. HCM patients were treated according to the latest guidelines for the diagnosis and management of HCM [17]. Additionally, 32.7% of HCM patients had a cardioverter defibrillator for the primary prevention of sudden cardiac death. The baseline characteristics of the patients with HCM are listed in Table 3. Treatment of the patients with HCM are presented in Table 4.

**Figure 1:**
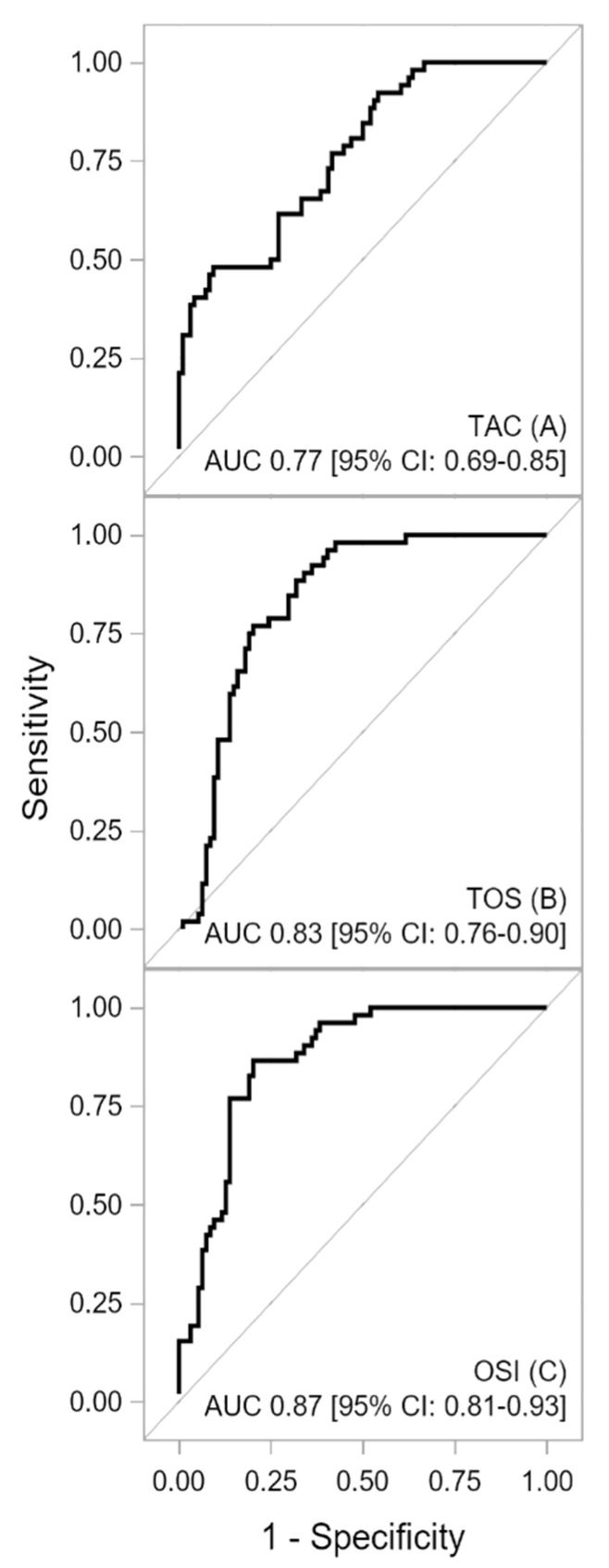
Receiver operating characteristic curves for TAC (**A**), TOS (**B**), and OSI (**C**) concentrations. Description: The area under the receiver operating characteristic curve indicated a good discriminatory power of TAC and TOS levels for HCM detection. The combined assessment of the oxidative-antioxidative balance by OSI showed excellent discriminatory power for HCM detection. Abbreviations: AUC—area under curve; CI—confidence interval; OSI—oxidative stress index; TAC—total antioxidant capacity, TOS—total oxidant status.

**Figure 2:**
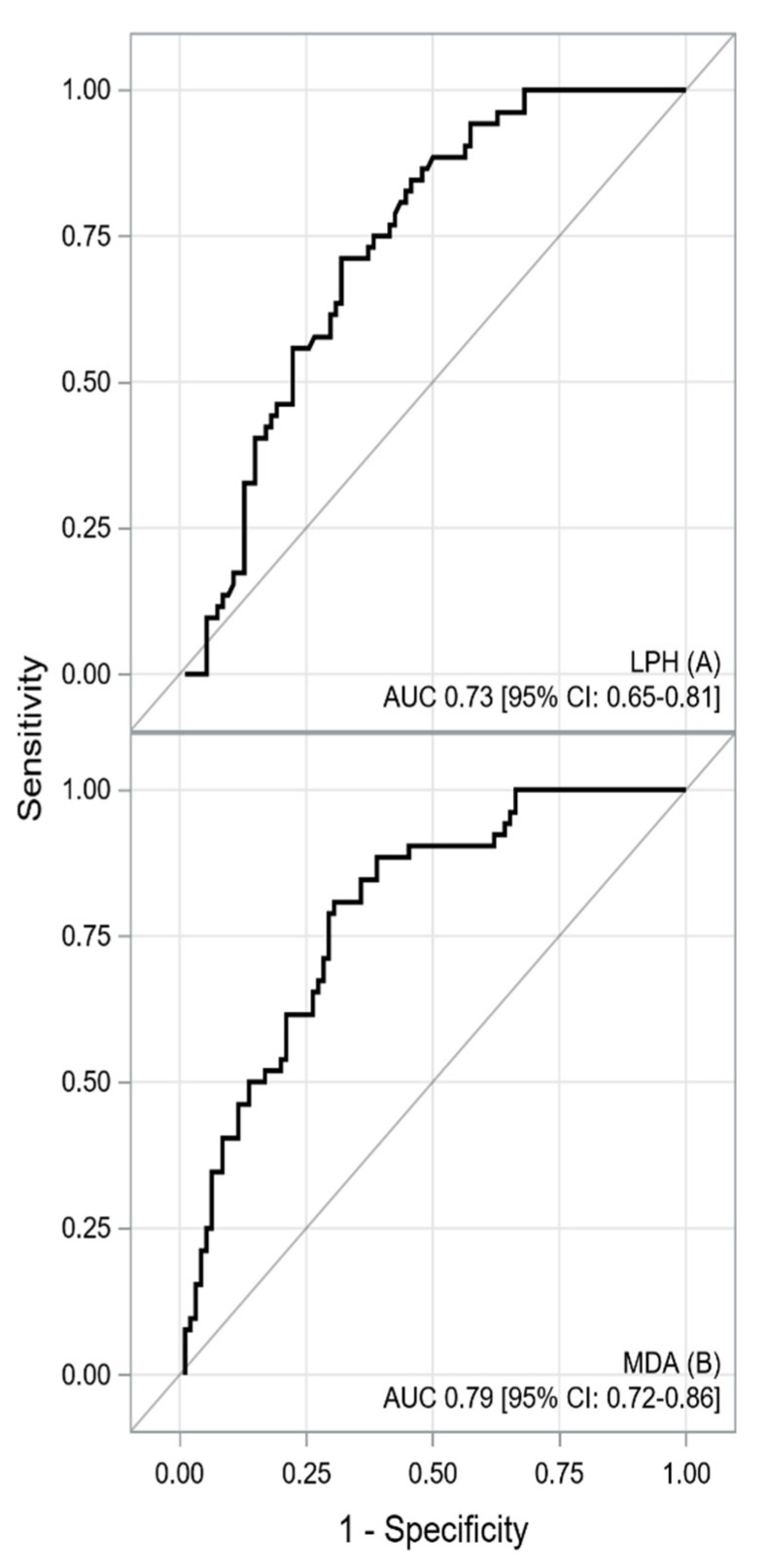
The receiver operating characteristic curve for LPH (**A**) and MDA (**B**) concentrations. Description: The area under the receiver operating characteristic curves indicated acceptable predictive power of LPH and MDA serum concentrations for HCM detection. Abbreviations: AUC—area under curve; CI—confidence interval, LPH—lipid hydroperoxide; MDA—malondialdehyde.

The ROC curves for oxidative-antioxidative parameters are shown in Figure 1 and Figure 2. The AUC indicated good discriminatory power of TAC and TOS levels for HCM detection. The combined assessment of TAC and TOS levels with OSI contributed to a significantly better detection of HCM (a better AUC, sensitivity, NPV, LR-, and accuracy). The cutoff value of ≥6.518 for OSI yielded a sensitivity of 87% and specificity of 80%. The difference between the calculated AUC for TAC and OSI levels amounted to 0.0986 (95% CI: 0.0317–0.1655) and that between the TOS level and OSI was 0.0372 (95% CI: 0.0042–0.0702), both of which were statistically significant (*p* = 0.004 and *p* = 0.0271, respectively). Furthermore, lipid peroxidation markers also generated acceptable power to detect HCM in patients [AUC_LPH_ = 0.73, *p* < 0.001; AUC_MDA_ = 0.79, *p* = 0.001]. Similarly, the difference between the AUCs for OSI and LPH [0.1377 (95% CI: 0.0731–0.2024), *p* < 0.001] was statistically significant. The difference between the AUCs for OSI and MDA amounted to 0.0782 (95% CI: 0.0056–0.1619), which was statistically nonsignificant (*p* = 0.0673). However, MDA showed worse sensitivity and specificity in terms of HCM detection than OSI (sensitivity 81% vs. 87%; specificity 70% vs. 80%, respectively). A summary of the results obtained from the ROC analysis for the oxidative-antioxidative biomarkers is presented in Table 5. 

**Figure 3:**
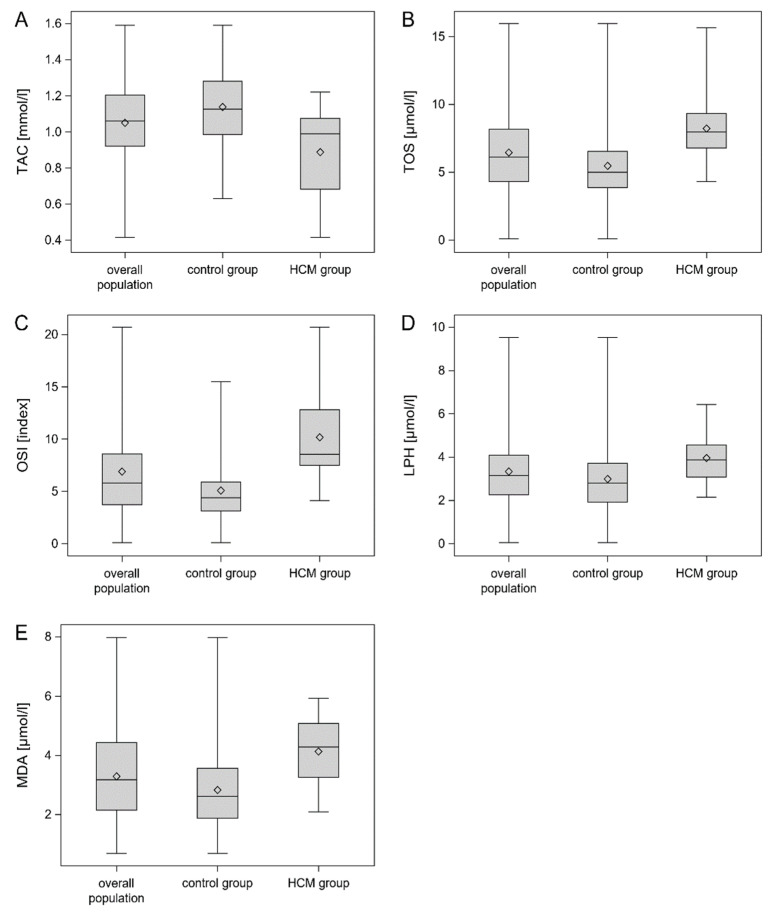
Box with whisker plots for TAC (**A**), TOS (**B**), OSI (**C**), LPH (**D**) and MDA (**E**) concentrations. Legend: Figure 3 presents box-and-whisker plots of the TAC, TOS, OSI, LPH and MDA serum concentrations in the total population, HCM group and control group. The concentrations of TOS, OSI, LPH and MDA were significantly higher in the HCM group than in the control group (*p* < 0.001). In turn, the TAC concentration was significantly lower in the HCM group than in the control groups (*p* < 0.001). The lower and upper lines of the “box” are the 25th and 75th percentiles of the sample. The distance between the top and bottom of the box is the interquartile range. Abbreviations: LPH—lipid hydroperoxide; MDA—malondialdehyde; OSI—oxidative stress index; TAC—total antioxidant capacity, TOS—total oxidant status.

Box with whisker plots of TAC, TOS, OSI, LPH and MDA serum concentrations are shown in Figure 3A–E. 

In the analyzed population, we observed significant correlations (negative and positive) between all analyzed oxidative stress markers. A summary of Spearman’s rank correlation coefficients between the analyzed parameters of oxidative stress is presented in Table 6.

## 4. Discussion

This study demonstrated a shift in the oxidative–antioxidative balance toward prooxidation in patients with HCM compared with control individuals. To the best of our knowledge, this is the first study assessing the total oxidative-antioxidative balance by measuring TAC, TOS, and OSI levels in circulating blood in an HCM patients population. Our analysis showed an increase in the secretion of ROS, identified by higher values of TOS and a decreased total activity of antioxidants, characterized by lower values of TAC, in patients with HCM than in those without HCM. TAC and TOS alone had good discriminatory power for HCM detection. The combined assessment of the oxidative-antioxidative balance by OSI showed excellent discriminatory power and good sensitivity and specificity for detecting HCM in the analyzed population. Furthermore, we found an association between the lipid peroxidation markers of LPH and MDA levels and the presence of HCM.

Oxidative stress occurs as a result of an imbalance between free radical production and endogenous antioxidant defense [13]. It is difficult to measure each antioxidant separately due to the number of different enzymatic and nonenzymatic antioxidants. Likewise, single oxidants do not reflect total exposure to oxidative stress. Therefore, TAC and TOS measurements have been used to assess oxidant and antioxidant systems in organisms [21,22,25]. In addition, OSI, which is the ratio of total plasma TOS to TAC, reflects the overall redox balance between oxidation and antioxidation, which makes it possible to estimate the total exposure to oxidative stress [13]. Oxidative-antioxidative imbalances are common mediators in the pathogenesis of many diseases related to the cardiovascular system [26]. However, relatively little is known about the role of oxidative stress in HCM. The studies conducted thus far on the role of oxidative stress in HCM patients have assessed the importance of individual markers, mainly lipid peroxidation indicators [8,14,27,28]. However, those studies confirmed a significantly higher level of oxidative stress markers in the HCM group than in the control group [8,14,27,28]. The presence of increased oxidative stress was also shown in an experimental model of myocardial hypertrophy associated with left ventricular pressure overload [28]. Pressure overload was observed in HCM patients with LVOT obstruction, generating a subvalvular gradient [27]. In turn, in the myocardium exposed to chronic pressure load, nitric oxide synthase 3 (NOS3) uncoupling was observed. NOS3 is considered a major source of myocardial ROS and is linked to hypertrophic remodeling [29]. Under physiological conditions, NOS3 synthesizes nitric oxide, which has an antihypertrophic effect. However, with pressure load, NOS3 is uncoupled, which is associated with a reduction in tetrahydrobiopterin-4 levels, the promotion of ROS production, and the activation of cardiac myocyte hypertrophy [29]. ROS production is derived not only from uncoupled nitric oxide synthases, but also from sources including mitochondria, xanthine oxidase, and nicotinamide adenine dinucleotide phosphate oxidases [17,26]. 

Another important source of ROS in HCM is mutant sarcomere proteins, which overproduce ROS through changes in cardiac performance and metabolism, mitochondrial activation or dysfunction, impaired protein quality control, and microcirculation dysfunction [17]. In turn, the disrupted redox balance on top of a sarcomeric gene mutation may contribute to HCM initiation and progression [17]. ROS act as signaling molecules, stimulating the activation of a broad variety of signaling kinases and transcription factors, which are associated with cardiac myocyte hypertrophy [18]. General oxidation of cells caused by ROS leads to dysfunction, hypertrophy, injury, apoptosis, and necrosis of cardiac myocytes. ROS also have an impact on molecular mechanisms, including the induction of specific posttranslational modifications that alter the function of important cellular proteins and signaling pathways in the heart, that can lead to hypertrophic remodeling of the heart [14,26,27,28]. ROS also play an important role in G protein-coupled hypertrophic stimulation by angiotensin II and tumor necrosis factor-α, as well as α-adrenergic stimulation [28,30,31]. In turn, cardiac myocyte hypertrophy induced by angiotensin II and tumor necrosis factor-α (TNF-α) can be inhibited by antioxidants [28,30,31]. Angiotensin II may contribute to cardiomyocyte hypertrophy through several intracellular pathways. These effects include the activation of protein kinase C, c-Jun N-terminal kinase, extracellular signal-regulated kinase, and ROS formation [30,32]. In turn, the role of TNF-α within the heart is not well known. However, it has been suggested that TNF-α may have an important autocrine or paracrine role in modulating myocardial homeostasis by means of activating matrix metalloproteinases, which are capable of degrading extracellular matrix syntax and promoting hypertrophic growth in cardiac myocytes [31]. However, cardiac myocyte hypertrophy induced by angiotensin II and TNF-α can be inhibited by antioxidants [28,30,31]. This fact may indicate a potential role of antioxidants in inhibiting the progression of cardiac hypertrophy.

Another interesting finding of the present study was the association between the lipid peroxidation markers of LPH and MDA and the presence of HCM. In our study, the levels of LPH and MDA were significantly higher in the HCM group than in the control group. LPH and MDA had acceptable predictive power, sensitivity, and specificity, allowing for the effective separation of HCM patients from control individual. The study by Dimitrov et al. also confirmed that HCM was associated with increased oxidative stress. This was characterized by an elevated level of the lipid oxidation product 8-iso-prostaglandin, which was the highest in the group of patients with LVOT obstruction [27]. In turn, the study by Nakamura et al. showed that 4-hydroxy-2-nonenal, which is a highly reactive diffusible product of lipid peroxidation, was significantly higher in endomyocardial biopsy samples from the patients with HCM compared to the control group, and their levels correlated with left ventricular dilatation and systolic dysfunction [30]. Another study also demonstrated that an increased release of ROS from mitochondria contributed to myocardial oxidative damage, and the level of lipid peroxides reflecting the severity of oxidative stress was significantly higher in the HCM group than in the control group [8]. The mechanisms whereby elevated serum markers of lipid peroxidation predict HCM are still not fully understood and remain unclear. From a pathophysiological point of view, lipids are particularly susceptible to oxidation due to their molecular structure containing reactive double bonds [33]. ROS cause damage to lipid membranes, including the sarcolemma, nucleus, sarcoplasmic reticulum, and mitochondrial membranes, leading to the formation of lipid radicals and LPHs [8,17,34]. Under normal physiological conditions, LPHs are detoxified by the restorative action of exogenous and endogenous antioxidants [35]. As the lipid peroxidation cascade progresses, LPH, the starting product, reacts with another fatty acid to form more stable late-stage products, including MDA or 4-hydroxy-2-nonenal [36]. Lipid peroxidation contributes to a change in the molecular structure of the lipid bilayer, causing damage and increasing the permeability of cardiomyocytes to ROS [17,33]. Destabilization of phospholipid-rich inner mitochondrial membrane integrity by lipid peroxidation leads to additional electron leakage and an intensification of the ROS production cascade [1,3]. In turn, damage to the mitochondrial membranes by ROS and mitochondrial oxidative stress are molecular mechanisms contributing to the development and progression of HCM [8]. 

The present study has several limitations. This is a single-center study involving a relatively small group of patients. Given the limitations of the study design, no causal relationship between the oxidative stress marker serum concentrations and HCM could be established. Therefore, it could not be clarified whether the increased level of oxidative stress markers and the decreased level of markers of antioxidant defense in HCM are the causative factors or a manifestation of the disease. The clinical utility of plasma levels of oxidative stress markers in the evaluation of cardiac hypertrophy in HCM remains to be established. To accurately determine the relationship between oxidative stress and HCM, oxidant-antioxidant balance markers in endomyocardial biopsy samples should be assessed. A further limitation of the study is the lack of an independent validation cohort that would support the role of oxidative stress markers in HCM. It is likely that the AUC for oxidative stress markers would have been lower if an independent validation cohort had been used. Because the study protocol of patients in the control group did not include echocardiography HCM cannot be ruled out with certainty in this group. However, a detailed history and the assessment of clinical features of the patients did not indicate the presence of this disease. Furthermore, given that the prevalence of hypertrophic cardiomyopathy in general population is 1:500 and the LIPIDOGRAM2015 & LIPIDOGEN2015 population consisted of consecutive primary care patients, the risk of HCM in this group is a population risk (1:500) and therefore should not significantly affect the conclusions of the study [37].

In addition, it should be emphasized that there are other conditions (apart from HCM) that may be associated with ROS release and could influence the interpretation of the data. 

## 5. Conclusions

In summary, our study demonstrates for the first time changes in the total oxidative–antioxidative balance as defined by TAC, TOS, and OSI in an HCM patient population. In our study, TAC and TOS levels had good discriminatory power for detecting HCM patients. The holistic assessment of oxidative stress by OSI had excellent discriminatory power and good sensitivity and specificity, and it could identify patients with HCM. Furthermore, another interesting finding of the present study is the association between the lipid peroxidation markers of LPH and MDA and the presence of HCM. LPH and MDA had acceptable predictive power, sensitivity, and specificity and allowed for the effective separation of HCM patients from control subjects.

## Figures and Tables

**Table 1:** Baseline characteristics of the patients.

	Control Group *N* = 97 ^#^	HCM Group *N* = 52 ^#^	*p* *
Age, years	52 (40–63)	52.5 (40–63.5)	0.927
Female, *n* (%)	57 (58.8)	30 (57.7)	0.8994
SBP, mmHg	130 (120–140)	125 (120–131.50)	0.1636
DBP, mmHg	80 (75–88)	80 (70–80)	0.119
Hypertension, *n* (%)	52 (53.6)	28 (53.8)	0.9779
Type 2 diabetes, *n* (%)	0 (0)	3 (5.8)	0.0077 *
Hypercholesterolemia, *n* (%)	51 (52.6)	34 (65.4)	0.1322
BMI, kg/m^2^	28.26 (4.12)	28.42 (4.46)	0.8286

^#^ Data are presented as medians (25th–75th percentile), means (standard deviation), or numbers (percentage) of patients. * *p* < 0.05 (statistically significant). Abbreviations: BMI—body mass index; DBP—diastolic blood pressure; SBP—systolic blood pressure.

**Table 2:** Markers of oxidative stress.

	Control Group *N* = 97 ^#^	HCM Group *N* = 52 ^#^	*p* *
TAC, mmol/L	1.13 (0.99–1.28)	0.99 (0.68–1.07)	<0.001 *
TOS, μmol/L	4.98 (3.86–6.53)	7.95 (6.78–9.32)	<0.001 *
OSI	4.38 (3.12–5.88)	8.54 (7.49–12.79)	<0.001 *
LPH, μmol/L	2.80 (1.91–3.71)	3.86 (3.07–4.55)	<0.001 *
MDA, μmol/L	2.61 (1.87–3.56)	4.28 (3.25–5.08)	<0.001 *

^#^ Data are presented as medians (25th–75th percentile), * *p* < 0.05 (statistically significant). Abbreviations: LPH—lipid hydroperoxide; MDA—malondialdehyde; OSI—oxidative stress index; TAC—total antioxidant capacity, TOS—total oxidant status.

**Table 3:** Baseline characteristics of patients with HCM.

Parameters	HCM Group *N* = 52 ^#^
Clinical features	
Chest pain	18 (34.6)
Dyspnoe	26 (50)
Syncope	21 (40.4)
Palpitations	19 (38.4)
Echocardiographic parameters	
SAM, *n* (%)	19 (36.5)
IVS max, mm	18.50 (16.00–21.00)
LVDD, mm	48.00 (43.00–52.00)
LVEF, %	55.50 (54.50–63.00)
LA, mm	43.00 (38.00–48.00)
RVDD, mm	36.00 (33.00–39.00)
TAPSE, mm	23.00 (21.00–26.50)
RVSP, mmHg	25.00 (16.50–25.50)
Ecg features	
LVH, *n* (%)	50 (96.1)
AF, *n* (%)	15 (28.8)
nsVT present, *n* (%)	26 (50)
HR mean/min	70.00 (63.00–74.00)
Laboratory parameters	
WBC, ×10^9^/L	6.47 (5.70–7.11)
Hemoglobin, mmol/L	8.90 (8.25–9.35)
Creatinine, µmol/L	80.00 (69.00–95.00)
Potassium, µmol/L	4.40 (4.16–4.61)
Sodium, mmol/L	141.00 (139.50–142.00)
Total bilirubin, µmol/L	10.30 (7.35–12.8)
NT-proBNP, pg/mL	622.50 (166.90–1171.00)
Other	
6MWT (m)	510.00 (455.00–575.00)

^#^ Data are presented as medians (25th–75th percentile), or numbers (percentage) of patients. Abbreviations: AF—atrial fibrillation; HCM—hypertrophic cardiomyopathy; HR—heart rhythm; LA—left atrium; LDL—low-density lipoprotein; LVEDD—left ventricular end-diastolic dimension; LVEF—left ventricular ejection fraction; LVH—left ventricular hypertrophy; LVOT—left ventricular outflow tract;; nsVT—non-sustained ventricular tachycardia; NT-proBNP—N-terminal prohormone of brain natriuretic peptide; RVDD—right ventricular diastolic dimension; RVSP—right ventricular systolic pressure; SAM—systolic anterior motion, TAPSE—tricuspid annular plane systolic excursion; WBC—white blood cells, 6MWT—six minutes walking test.

**Table 4:** Pharmacological treatment of patients with HCM.

Parameters	HCM Group (*N* = 52) ^#^
Beta-blokers, *n* (%)	46 (88.5)
Cordarone, *n* (%)	3 (5.8)
ACEI/ARB, *n* (%)	26 (50)
MRA, *n* (%)	13 (25)
Verapamil, *n* (%)	6 (11.5)
Statin, *n* (%)	28 (53.8)

^#^ Data are presented numbers (percentage) of patients. Abbreviations: ACEI—angiotensin coverting enzyme inhibitor; ARB—angiotensin II receptor blocker, MRA—mineralocorticoid receptor antagonists.

**Table 5:** A summary of the ROC curve analysis for the analyzed biomarkers.

	AUC [±95 CI]	*p*	Cut-Off	Sensitivity [±95 CI]	Specificity [±95 CI]	PPV [±95 CI]	NPV [±95 CI]	LR+ [±95 CI]	LR− [±95 CI]	Accuracy
TAC	0.7674 (0.6903–0.8445)	<0.0001	<0.91	48 (34–62)	90 (82–95)	71 (54–85)	0.76 (0.67–0.84)	4.66 (1.61–7.72)	0.58 (0.42–0.74)	0.75 (0.67–0.82)
TOS	0.8304 (0.7642–0.8966)	<0.0001	≥6.689	77 (63–87)	80 (71–88)	68 (54–79)	68 (54–0.79)	3.93 (2.23–5.63)	0.29 (0.14–0.43)	0.79 (0.72–0.85)
OSI	0.8676 (0.8103–0.9250)	<0.0001	≥6.518	87 (74–94)	80 (71–88)	70 (58–81)	92 (84–97)	4.42 (2.56–6.27)	0.17 (0.05–0.28)	0.83 (0.75–0.88)
LPH	0.7316 (0.6515–0.8117)	<0.0001	≥3.264	71 (57–83)	69 (59–78)	55 (43–67)	82 (72–89)	2.30 (1.50–3.1)	0.42 (0.23–0.61)	0.70 (0.62–0.77)
MDA	0.7917 (0.7190–0.8644)	<0.0001	≥3.198	81 (67–90)	70 (60–79)	59 (47–71)	87 (78–94)	2.70 (1.80–3.61)	0.27 (0.12–0.43)	0.74 (0.66–0.81)

Abbreviations: see Table 1; LR—negative likelihood ratio; LR+—positive likelihood ratio; NPV—negative predictive value; PPV—positive predictive value; ROC—receiver operating characteristic.

**Table 6:** Spearman’s rank correlation coefficients between analyzed parameters of oxidative–antioxidant balance in the analyzed population.

Variable	TOS, umol/L	TAC, mmol/L	OSI	LPH, ummol/L	MDA, umol/L
TOS, umol/L	1.000	−0.422 *	0.932 *	0.813 *	0.491 *
TAC, mmol/L	−0.422 *	1.000	−0.677 *	−0.359 *	−0.387 *
OSI	0.932 *	−0.677 *	1.000	0.776 *	0.509 *
LPH, umol/L	0.813 *	−0.359 *	0.776 *	1.000	0.433 *
MDA, ummol/L	0.491 *	−0.387 *	0.509 *	0.433 *	0.491 *

Abbreviations: see Table 1. * *p* < 0.001.

## Data Availability

The data presented in this study are available upon request from the corresponding author.
